# A blade-free approach to the Tzanck smear

**DOI:** 10.1016/j.jdin.2024.02.011

**Published:** 2024-04-04

**Authors:** Ananya Sharma, Rajat Choudhary, Japnoor Kaur, Ridhi Sood, Somesh Gupta

**Affiliations:** a Department of Dermatology and Venereology, All India Institute of Medical Sciences, New Delhi, India; b Department of Pathology, All India Institute of Medical Sciences, New Delhi, India

**Keywords:** autoimmune bullous diseases, bedside test, cytology, diagnostics, pemphigus, Tzanck smear

## Challenge

Tzanck smear is a simple, rapid, and minimally invasive bedside cytodiagnostic technique used for vesiculobullous or erosive lesions. Although histopathology and direct immunofluorescence are the gold standard for pemphigus group of disorders, Tzanck smear is a cost-effective tool in resource-poor settings, with a sensitivity of 80.5% and specificity of 84.6% for oral pemphigus.^1^ However, the standard procedure in dermatology literature involves scraping the erosion with the blunt end of a surgical blade, often causing patient apprehension, pain, and bleeding. Moreover, lesions on the posterior part of buccal mucosa or palate are inaccessible by this method (Supplementary Fig 1, available via Mendeley at https://data.mendeley.com/datasets/ks2gwtckh3/1).

## Solution

**Fig 1:**
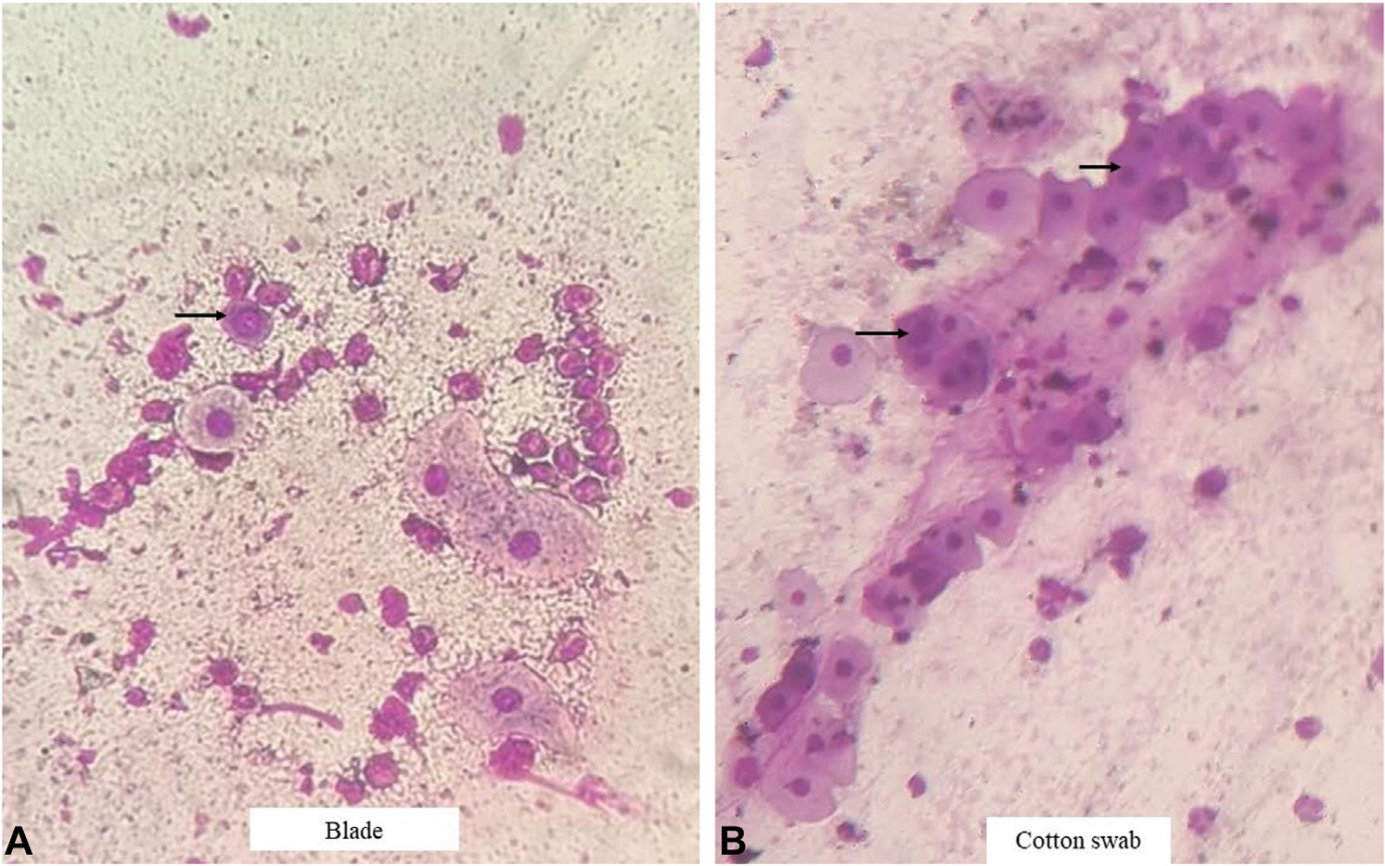
Tzanck smear made with (**A**) the traditional blade method compared with that made with (**B**) a cotton swab. Black arrows show acantholytic cells, seen in both smears. Individual cell morphology is better in the smear made with a cotton swab, along with fewer artifacts.

Vaginal swabs from intact mucosa are universally prepared using cotton swabs and yield adequate epithelial cells. Extending this observation, we prepared Tzanck smears using dry cotton swabs gently rolled over the lesion from oral erosions of 6 patients with pemphigus. When compared with smears made using a surgical blade (Fig 1, *A*), individual cell morphology was better appreciated in swab smears, with fewer artifacts (Fig 1, *B*; Supplementary Fig 2, available via Mendeley at https://data.mendeley.com/datasets/ks2gwtckh3/1). However, the cell yield with swabs was slightly lower than that with the blade method. All patients uniformly reported feeling significantly less apprehensive with the use of a cotton swab, although objective pain scores were similar. It was also possible to make smears demonstrating acantholytic cells from erosions on the palate and posterior part of buccal mucosa that could not be scraped using a blade. Polyester swabs showed similar yield. This method can also be used as a noninvasive, patient-friendly method for cellular yield for direct immunofluorescence (Supplementary Fig 3, available via Mendeley at https://data.mendeley.com/datasets/ks2gwtckh3/1).^2^

## Conflicts of interest

None disclosed.
